# The globalization of naval provisioning: ancient DNA and stable isotope analyses of stored cod from the wreck of the Mary Rose, AD 1545

**DOI:** 10.1098/rsos.150199

**Published:** 2015-09-09

**Authors:** William F. Hutchinson, Mark Culling, David C. Orton, Bernd Hänfling, Lori Lawson Handley, Sheila Hamilton-Dyer, Tamsin C. O'Connell, Michael P. Richards, James H. Barrett

**Affiliations:** 1 Evolutionary Biology Group, Department of Biological Sciences, University of Hull, Hull HU6 7RX, UK; 2 BioArCh, Department of Archaeology, University of York, York YO10 5DD, UK; 3 SH-D ArchaeoZoology, 5 Suffolk Avenue, Shirley, Southampton SO15 5EF, UK; 4 McDonald Institute for Archaeological Research, Department of Archaeology and Anthropology, University of Cambridge, Cambridge CB2 3ER, UK; 5 Department of Anthropology, University of British Columbia, Vancouver Campus, 6303 NW Marine Drive, Vancouver, British Columbia, Canada V6T 1Z1; 6 Department of Human Evolution, Max Planck Institute for Evolutionary Anthropology, Deutscher Platz 6, 04103 Leipzig, Germany

**Keywords:** historical ecology, single-nucleotide polymorphisms, stable isotope analysis, cod, fish trade, Mary Rose

## Abstract

A comparison of ancient DNA (single-nucleotide polymorphisms) and carbon and nitrogen stable isotope evidence suggests that stored cod provisions recovered from the wreck of the Tudor warship Mary Rose, which sank in the Solent, southern England, in 1545, had been caught in northern and transatlantic waters such as the northern North Sea and the fishing grounds of Iceland and Newfoundland. This discovery, underpinned by control data from archaeological samples of cod bones from potential source regions, illuminates the role of naval provisioning in the early development of extensive sea fisheries, with their long-term economic and ecological impacts.

## 1. Introduction

Historical ecology has become an essential step in understanding the long-term human exploitation of aquatic ecosystems [1,2]. In one important example, the growth of long-range trade in high-bulk staple products in medieval and post-medieval Europe underpinned the development of urbanized market economies, colonialism, empires and concomitant environmental impacts [3–5]. Concurrent with conquest and deforestation for increased cash-crop production, these periods saw the expansion of extensive sea fishing. Historical research, zooarchaeological evidence and stable isotopic analysis of archaeological fish bones all suggest that preserved Arctic Norwegian and North Atlantic cod were increasingly transported to consumers around the North Sea—particularly expanding urban populations—between the eleventh and sixteenth centuries [6–9]. An open question in this context is whether the requirements of naval provisioning may also have played a role in the development of extensive sea fisheries and, concurrently, whether the availability of preserved fish from distant seas helped sustain Europe's first standing navies. This question is especially pertinent for the sixteenth century, which saw both the naissance of European transatlantic colonialism and the growing importance of sea power as a tool in increasingly global conflicts [10].

An unparalleled opportunity to investigate the role of fish in early naval provisioning is provided by the wreck of the Tudor warship Mary Rose, which sank in the Solent, southern England, in 1545 while sailing to military action with a full complement of crew and a full store of provisions [11]. Among the excavated remains of its supplies were thousands of cod bones, some associated with casks and baskets. The find context and absence of cranial bones strongly suggest that they were from dried or salted cod, staples of the Tudor naval diet, but where had they been caught? New stable isotopic methods provide a promising way to detect non-local imports of cod [7,12,13] and genetic markers have proved useful for investigating population differentiation in marine fishes. Single-nucleotide polymorphisms (SNPs) are especially well suited for identifying source populations using DNA from archaeological samples, because they combine the power to detect even weak structuring on a small geographical scale [14–16] with their utility for genotyping highly degraded ancient DNA samples (less than 100 bp). Because the stable isotope data employed (*δ*^13^C and *δ*^15^N of bone protein) reflect diet and local environmental conditions [17], whereas genetic markers reflect genetic drift, gene flow and selection [18], the methods are independent. Thus, together they can provide complementary information on the potential source of traded fish.

Prior to the use of ice or refrigeration for shipping fresh foods, fish such as cod, *Gadus morhua*, an important commercial species since the Middle Ages, were typically (although not exclusively) decapitated before drying and/or salting for long-distance transport [19,20]. Consequently, stable isotope and DNA signatures recovered from archaeological cranial bones of cod can be used to provide a baseline set of control data to which potentially transported post-cranial target bones of this species can be assigned. In this way, the development of long-distance trade, and/or long-range fishing trips, can be investigated.

The undocumented suppliers to the Mary Rose could have drawn on preserved cod from a diverse, but probably finite, number of regions. England's salt cod fisheries were well developed by the sixteenth century, involving both relatively local inshore catches (particularly around the southwest coast and in the North Sea) and long-distance fisheries conducted off southern and western Ireland, Iceland and (from *ca* 1502) northeastern North America, particularly Newfoundland [21–23]. Additional possibilities include dried and salted cod from northern Scotland [20,24] and Norway [25]. Lastly, stockfish (cod dried without salt, especially from Norway and Iceland), which had dominated earlier medieval trade, was also still available [21,22]. Were cod provisions on the Mary Rose caught locally or sourced from some of these distant waters? If the latter, from which population or populations? This paper aims to answer these questions by analysing SNP genotypes and stable isotope signatures using a set of control samples (*n*=168 and 239, respectively) from potential source locations and comparing these with 11 cod bones from the Mary Rose. The results contribute to our understanding of globalization during the transition from the European Middle Ages to the era of transatlantic expansion. In particular, they address the role of distant food sources in the provisioning of a standing navy.

## 2. Material and methods

### 2.1 Control samples

Both aDNA and stable isotope analyses require baseline data for cod bones from potential source populations (controls) in order to attribute the Mary Rose samples (targets) to possible regions of catch. Natural and anthropogenically induced temporal changes in the baseline signatures are known to occur, particularly with recent variations in genetic selective pressures (e.g. due to fluctuating sea temperatures and the development of industrial fishing) and increasing isotopic pollution due to farming (with its chemical runoff) and industrialization [26–30]. Thus, pre-modern samples were required. On the basis that most cod were decapitated prior to drying, we used cranial bones from archaeological sites as proxies for relatively local catches within a series of potential source regions. Contemporaneity with the Mary Rose across regions was not an achievable objective given the vagaries of the archaeological record, and the control data were gathered as part of a wider study to include earlier centuries, but in all cases the chronology of the control samples predates industrial fishing, farming and manufacturing in the region in question. Earlier natural variation, owing to climate change for example [29], and localized spatial shifts in cod distribution due to varying water temperatures [31], may reduce the discriminating power of our methods, but is unlikely to invalidate them (see below).

**Figure 1.**
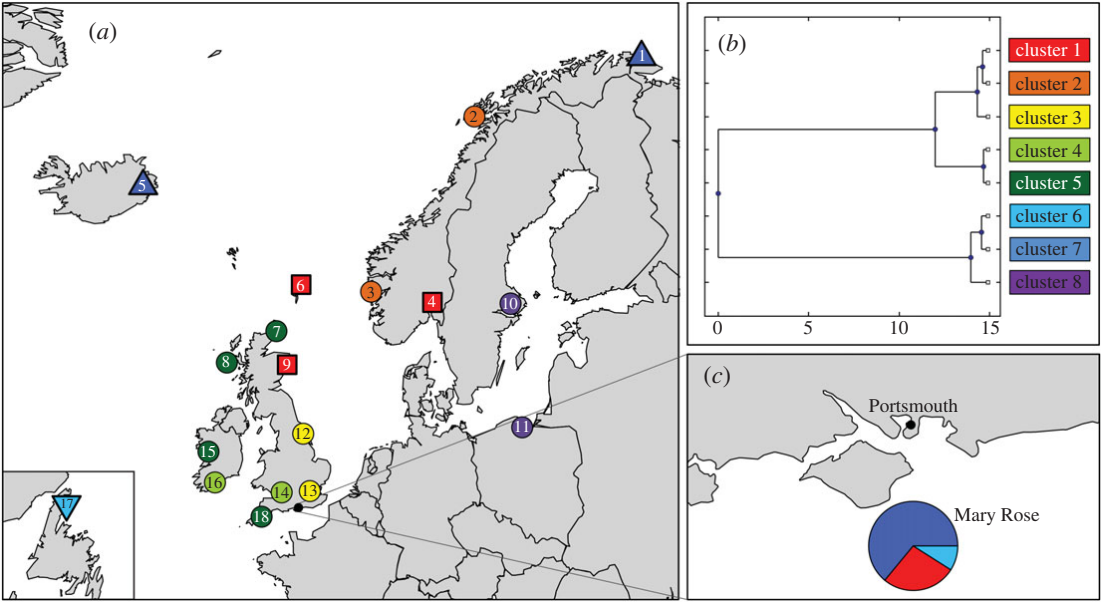
Genetic samples: (*a*) locations of control samples (the squares and triangles indicate clusters to which the target samples were assigned; see table 1 for key to site numbers); (*b*) UPGMA dendrogram of Kullback–Leibler divergence showing relationship between the eight genetic clusters of control data; (*c*) the proportion of Mary Rose targets assigned to each genetic control cluster by BAPS.

We collected a geographically wide-ranging set of archaeological control samples of cod bone (table 1). In some cases, we were able to use samples from the same location (or even the same individual bones) for both aDNA and stable isotope analyses (electronic supplementary material, table S1). For aDNA, 19 collections of control skull bones (totalling 168 specimens with successful results) were obtained from archaeological excavations sited around the shores of the Barents Sea; the northern, western and southern Norwegian coast; the waters surrounding Britain and Ireland; the Baltic Sea; Iceland; and Newfoundland (figure 1*a*). Where multiple bones were sampled from the same archaeological context, these were either of the same anatomical element and side or clearly different in size, to eliminate the possibility of replicating results from individual fish. The genetic control samples range in date from the twelfth–thirteenth centuries to the eighteenth–nineteenth centuries (table 1). The use of comparatively recent eighteenth–nineteeth century control samples from Newfoundland was justified, as an SNP analysis of modern samples from across the cod's distribution (using the 28 loci described in this study) showed that 50 cod from Newfoundland (collected in 2011 at 48.16 latitude, −53.72 longitude) were highly divergent from populations in the east [32] where the rest of the control specimens were sourced (Arlequin v. 3.1, *θF*_ST_=0.279–0.774, *p*≤0.00001).

**Table 1.** The genetic and stable isotope control and target samples used in the study (further information is provided in the electronic supplementary material, table S1).

	aDNA		stable isotopes					
settlement	no.	date	no.	date	latitude	longitude	location	key to maps
Skonsvika	4	13th–14th	4	13th–14th	70.87	29.00	Arctic Norway	1
Kongshavn	6	14th	6	14th	70.85	29.21	Arctic Norway	1
Storvågan	8	12th–15th	5	12th–15th	68.20	14.45	Arctic Norway	2
Bergen	5	14th	—	—	60.40	5.32	western Norway	3
Oslo	11	12th–14th	—	—	59.92	10.73	southeast Norway	4
Skriðuklaustur	12	15th–16th	14	15th–16th	65.10	−14.82	Iceland	5
Sandwick	5	13th–14th	10	12th–14th	60.70	−0.87	northern Scotland	6
Robert's Haven	6	13th/14th	1	13th–14th	58.65	−3.05	northern Scotland	7
Bornais	9	12th–13th	10	12th–13th	57.25	−7.43	northern Scotland	8
Aberdeen	10	*ca* 13th–14th	4	*ca* 13th–14th	57.15	−2.10	northern Scotland	9
Uppsala	7	13th–14th	12	13th–15th	59.86	17.64	eastern Sweden	10
Gdask	7	13th–14th	11	13th–16th	54.36	18.66	northern Poland	11
York	13	13th–14th	3	10th–13th	53.96	−1.08	eastern England	12
London	10	13th–14th	16	8th–17th	51.51	−0.10	southeast England	13
Bristol	14	13th–14th	5	12th–14th	51.45	−2.59	western England	14
Galway	13	13th–14th	4	13th–14th	53.27	−9.05	western Ireland	15
Cork	10	medieval	3	medieval	51.90	−8.48	southern Ireland	16
Dos de Cheval	13	18th–19th	5	18th–19th	50.91	−55.87	Newfoundland	17
Launceston	5	13th–14th	—	—	50.37	−4.36	southwest England	18
Måsøy	—	—	10	17th–19th	70.98	24.63	Arctic Norway	19
Helgøygården	—	—	6	14th	70.11	19.35	Arctic Norway	20
Vannareid	—	—	12	17th–19th	70.20	19.60	Arctic Norway	20
Quoygrew	—	—	34	11th–15th	59.34	−2.98	northern Scotland	21
Knowe of Skea	—	—	3	12th–16th	59.26	−2.98	northern Scotland	21
Carrickfergus	—	—	4	*ca* 16th–17th	54.72	−5.81	Northern Ireland	22
Dublin	—	—	9	medieval	53.34	−6.27	eastern Ireland	23
Waterford	—	—	2	medieval	52.26	−7.11	southern Ireland	24
Wharram Percy	—	—	2	13th–14th	54.07	−0.69	eastern England	12
Norwich	—	—	6	11th–18th	52.63	1.30	eastern England	25
Cambridge	—	—	1	14th	52.21	0.12	eastern England	26
Southampton	—	—	9	9th–14th	50.90	−1.40	southern England	27
Exeter	—	—	2	11th–15th	50.72	−3.53	southwest England	28
Norden	—	—	1	13th–14th	53.60	7.20	northwest Germany	29
Mała Nieszawka	—	—	7	14th–15th	52.99	18.55	Poland	30
Raversijde	—	—	2	15th	51.20	2.85	Belgium	31
Mechelen	—	—	1	16th	51.03	4.48	Belgium	32
St John's	—	—	15	18th	47.56	−52.71	Newfoundland	33

**Figure 2.**
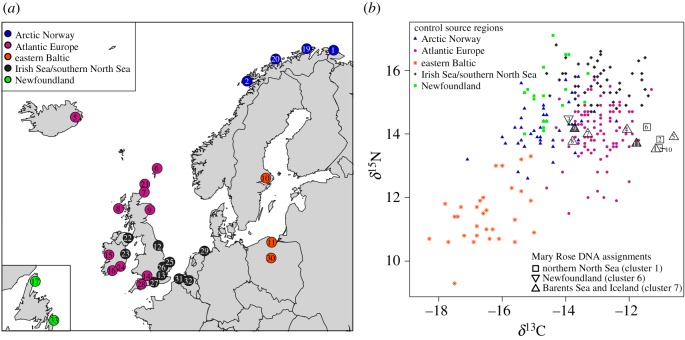
Stable isotope samples: (*a*) locations of control samples (see table 1 for key to site numbers); (*b*) scatterplot of stable isotope data for the five control macro-groups and the Mary Rose target samples (the Mary Rose sample symbols indicate the genetic cluster assignments; with the shaded examples indicating fish of greater than 1000 mm total length).

For stable carbon and nitrogen isotope analysis, 33 collections of control bones (totally 239 specimens with successful results) have been used (table 1). Data from Barrett *et al*. [7] are augmented by the addition of 55 new specimens from previously covered regions and 29 specimens from the formerly unsampled coasts of Ireland and southwestern Britain (figure 2). The latter regions developed commercial salt cod industries starting in the late fourteenth century based on historical evidence [23] and are potential *a priori* candidates for the source of fish provisions on a vessel sailing from Portsmouth. The stable isotope control data are all from cod with estimated total lengths (TL) of 500–1000 mm (based on bone measurements and/or comparison with reference specimens of known size, the former using established regression formulae [33] and the latter using 1:1 scanned images to avoid contamination) to minimize possible trophic-level effects on the isotope values [7]. The samples range in date from the late eighth to the early nineteenth centuries. The *δ*^13^C and *δ*^15^N values of marine fish are known to be influenced by water temperature, salinity, nutrient loading and the structure of the food web, all of which vary through time [12]. Nevertheless, when subdivided by region and date the only statistically significant difference between time periods in any of our pre-modern *δ*^13^C or *δ*^15^N datasets occurs in eastern Baltic *δ*^13^C (electronic supplementary material, table S2 and figures S1–S2). Moreover, this spatial group remains isotopically distinct from others regardless of the change through time (see below). Thus, based on present evidence, chronological fluctuations in environmental variables are most likely to be expressed as heterogeneity within the stable isotope data of each region, with concomitant limits on their resolution.

### 2.2 Target samples

A collection of 11 target samples of potentially traded cod cleithra were obtained from the excavation of the Mary Rose. Subsamples of the same specimens were used for both aDNA and stable isotope analyses. They were selected from 4384 identified fish bones (almost all of cod) originally recovered during excavation and the subsequent sieving of sediment samples. Only appendicular skeletal elements (such as the cleithrum which supports the pectoral fin behind the head) and vertebrae were present. There were no cranial elements. This anatomical pattern, combined with distinctive cut marks and the find contexts (e.g. associated with casks and baskets), strongly suggests that the bones were from stored cod, dried with or without salt [34]. Four of the target specimens analysed here (nos. 1, 3, 10 and 11) were from the stern of the ship in the hold, one of which (no. 3) was associated with a wicker basket. Six (nos. 4, 5, 6, 7, 8 and 9) were from the orlop deck above, two of which (nos. 5 and 6) were associated with a staved container or cask. One target specimen (no. 2) does not have a specific location recorded, but was nevertheless sealed within the wreck.

### 2.3 Genetic analysis

To determine the optimal loci for assigning the target samples, SNP data for 102 loci from a genetic analysis of contemporary samples [32], which replicated the potential geographical distribution of the archaeological control samples, were analysed to identify diagnostic loci for detecting spatial genetic structuring and assigning individuals to their source population. A total of 28 SNP loci were identified as the most informative for identifying population structuring and individual assignment, based on their high genetic divergence (*θF*_ST_) between the geographically distributed samples. These 28 loci were used in the subsequent ancient DNA analyses.

All ancient DNA laboratory work was carried out in a dedicated aDNA facility at the University of Hull with restricted access, and negative controls were used throughout the extraction and DNA amplification process. Each specimen was first cleaned of any soil particles with double-distilled water and subdivided in a disposable polythene chamber using a sterile fixed-blade scalpel. Approximately 1 g of bone was then decontaminated for genetic analysis according to Yang *et al.*'s [35] method. Briefly, the samples were immersed in 10% (w/v) bleach for 20 min, rinsed thoroughly in double-distilled water three times to remove the bleach, quickly immersed in 1 M hydrochloric acid and then 1 M sodium hydroxide to neutralize the acid, and finally rinsed a further three times in double-distilled water. The cleaned sample was then UV irradiated on each side for 20 min.

The dry bone was subsequently ground into a fine powder using a liquid nitrogen freezer mill, and 0.5 g of bone digested overnight in 9 ml of lysis buffer at 56°C [35]. The resulting solution was centrifuged for 5 min at 9500*g* to pellet the undigested material, and 8 ml of supernatant was treated with an inhibitEX tablet (Qiagen) to remove potential polymerase chain reaction (PCR) inhibitors, prior to a further centrifugation for 5 min at 9500*g*. In total, 2 ml of the supernatant was transferred to two Vivacon 2 micro-concentrators (30 kDa MWCO, Sartorius Stedim Biotech) and centrifuged at 2500*g* to concentrate the DNA, intermittently topping up the columns until 625 μl of supernatant remained in each. The two supernatants were subsequently combined and cleaned by passing through a QIAquick column (Qiagen), with 100 μl of DNA being eluted off the columns [35]. The solutions and columns were maintained at 56°C throughout the latter two stages to facilitate faster filtration. The aDNA was subsequently PCR-amplified using the selection of 28 informative SNP loci in four multiplex reactions, with each reaction containing seven different pairs of SNP primers (electronic supplementary material, table S3). The 50 μl PCRs contained 1× Qiagen Multiplex Mix, 0.2 μM of each primer, 0.1 mg ml^−1^ bovine serum albumin, RNase-free water, and 1 μl DNA extraction. A two-stage amplification, 36-cycle, *Touch-Down* PCR protocol was used to amplify the DNA, where the annealing temperature was reduced by 1°C in each cycle during the first stage of amplifications: 1. Initial denaturation at 95°C for 15 min. 2. First amplification using 10 cycles of 94°C for 20 s, 60→50°C for 90 s, 72°C for 45 s. 3. Second amplification using 26 cycles of 94°C for 20 s, 50°C for 90 s, 72°C for 45 s. 4. Final extension of 72°C for 30 min.

The strength of the resulting PCR products was assessed by agarose gel electrophoresis, prior to SNP genotyping using KBiosciences's KASPar assay. KASPar is a fluorescence-based competitive allele-specific PCR genotyping system (for a description of the technique, see http://www.lgcgenomics.com/genotyping/kasp-genotyping-chemistry). Ten per cent of the samples were re-amplified and re-genotyped to test for reproducibility.

### 2.4 Stable isotope analysis

Collagen was extracted and analysed for the stable carbon and nitrogen isotope ratios following the procedures reported by Barrett *et al*. [7]. A complete cross section (*ca* 100–200 mg) of each specimen was processed. Samples were demineralized in 0.5 M hydrochloric acid at 4°C for 2–5 days and then gelatinized in a solution of acidic (pH 3) water at 70°C for 48 h, with the resulting solution filtered through a 5–8 μm Ezee' filter (Elkay). The gelatinized solution was then ultrafiltered through a 30 kDa filter, and the greater than 30 kDa fraction lyophilized for 48 h. The resultant ‘collagen’ was analysed in duplicate or triplicate by continuous-flow isotope-ratio-monitoring mass spectrometry. A Thermo Finnigan Flash EA coupled to a Thermo Finnigan Delta Plus XP mass spectrometer was used at the Department of Human Evolution, Max Planck Institute for Evolutionary Anthropology, Leipzig, Germany, and a Costech EA coupled to a Thermo Finnigan Delta V Plus mass spectrometer at the Godwin Laboratory, Department of Earth Sciences, University of Cambridge. Electronic supplementary material, table S1, provides the results and indicates where the sample preparation and mass spectrometry were done (in Leipzig or Cambridge). Following convention, the carbon and nitrogen isotopic data are reported on the *δ*-scale in units of parts per thousand or ‘permil’ (‰), with *δ*^13^C values reported relative to V-PDB, and *δ*^15^N values relative to AIR [36,37]. Repeated measurements on international and in-house standards showed that the analytical error was less than 0.2‰ for both the *δ*^13^C and *δ*^15^N measurements. All reported samples produced acceptable atomic C:N ratios, defined as between 2.9 and 3.6 [38,39], indicating that the results are likely to reflect *in vivo* values. Nine of the target samples were from cod of the same size (TL) range as the control specimens. Two narrowly exceeded this size, but nevertheless had *δ*^13^C and *δ*^15^N values within the range of the other target specimens.

### 2.5 Statistical analysis of genetic data

Geographical structuring among control samples was investigated using a Bayesian maximum-likelihood approach as implemented in BAPS v. 5 [40]. The option ‘population based clustering’ was used to explore the separation of control samples into a range of genetic clusters, and to identify the most likely number of clusters (*K*) in the dataset. Support for the identified clusters was further tested using an analysis of molecular variance (AMOVA) in Arlequin v. 3.1 [41] to quantify the amount of variation partitioned within and between the clusters. An UPGMA dendrogram of Kullback–Leibler divergence estimates was plotted showing the genetic relationship between the identified clusters in BAPS v. 5.

The genetic clusters (i.e. groups of control samples) identified under the most suitable *K* were used as assignment units for individual target samples. A Bayesian maximum-likelihood based ‘Trained’ clustering technique (BAPS v. 5) was used to estimate the likelihood of each target sample being assigned to each of the identified clusters.

### 2.6 Statistical analysis of isotopic data

The control data were initially assigned to nine source regions based on geographical proximity and historically known fishing grounds: eastern Baltic Sea, Arctic Norway, northeast North Atlantic (Iceland and northern Scotland), Irish Sea, Irish west coast, Celtic Sea, eastern English Channel, southern North Sea and northwest Atlantic (Newfoundland). Where these regions proved indistinguishable based upon the observed carbon and nitrogen isotopic ratios (using linear discriminant analysis (LDA); see below) they were combined into broader groups that appear to reflect differences between open ocean and more enclosed waters around the British Isles: northeast North Atlantic, Irish west coast and Celtic Sea were merged into ‘Atlantic Europe’, while Irish Sea, (eastern) English Channel and southern North Sea became ‘Irish/southern North Seas’. This is a conservative approach, trading off reduced geographical resolution for increased confidence in our source predictions, while maintaining the geographical/hydrological coherence of our control groups so far as is possible.

A single extreme outlier was removed from the dataset: specimen 1554 from Carrickfergus produced *δ*^13^C and *δ*^15^N values of −12.5 and 12.7, respectively, giving *p*-values of less than 0.000001 based on membership both of the original Irish Sea source region and of the combined Irish/southern North Seas group (based on Mahalanobis distance from group centroids—*D*^2^=46.21 and 28.29, respectively). This specimen is very likely to represent either an individual from the Atlantic rather than the Irish Sea or measurement error. All other specimens were included in the final analysis.

For each of the Mary Rose target specimens, probabilities of membership of each of the five resulting control groups were calculated using LDA. This was performed in R 3.1.3 using the ‘lda’ and ‘predict.lda’ functions (MASS package v. 7.3-39) [42], with prior group membership probabilities (‘prior’) set to uniform and using leave-one-out cross-validation (‘CV=TRUE’) to evaluate the model, but otherwise default arguments.

## 3. Results

### 3.1 Genotyping of samples

Of the ancient samples that were extracted and genotyped at the 28 loci, 77% of the 168×28 control PCRs and 90% of the 11×28 target PCRs yielded informative genotypes, with no correlation between the age and the genotyping success rates (see the electronic supplementary material, table S4, for full genotype data). It was evident that the samples recovered from the Mary Rose excavation yielded substantially better DNA with a greater PCR success rate, which was likely due to the anoxic marine silt from which they were recovered being a more optimal preservation medium [34]. There was no clear sample-specific or geographical bias to the failed PCRs, and since multiple loci were used in this analysis, any bias introduced by individual weakly amplifying loci is likely to have a very marginal impact on estimates of population structuring.

Repeated genotyping of the samples identified the presence of allelic dropout (i.e. only one allele is amplified in heterozygotes) during PCR amplification in 14% of the control genotypes (66 PCRs), while the target samples showed consistently repeatable results. However, the loss of alleles was random, and since the genetic assignment methods used were based upon allele frequency rather than estimated heterozygosity, the only impact was to make the assignment of the target samples more conservative.

### 3.2 Cluster analysis of genetic baseline control data

The Bayesian maximum-likelihood analysis grouped the 19 sample groups into eight genetic clusters (table 2). A complementary AMOVA within and between these eight clusters indicated that there was no significant variation among populations within clusters (table 3). The relative genetic distance between the eight clusters is illustrated by the UPGMA dendrogram of the Kullback–Leibler divergence estimates (figure 1*b*).

**Table 2.** Assignment of the 19 control populations into eight clusters with BAPS. Values are the change in log (marginal likelihood) if a sample is moved from its most likely cluster (logML=0) to a different cluster.

		cluster							
cluster name	archaeological source	1	2	3	4	5	6	7	8
northern North Sea	Aberdeen	0	−27.2	−43.2	−43.8	−80.6	−140.5	−119.8	−89.3
	Sandwick, Shetland	0	−33.4	−39.6	−43.6	−59.3	−112.4	−73.9	−47.4
	Oslo	0	−9.7	−16.9	−35.7	−115.7	−116.8	−78.3	−59.6
Lofoten and Bergen	Storvågan, Lofoten	−7.7	0	−6.2	−36.3	−119.0	−66.0	−52.2	−35.6
	Bergen	−13.7	0	−8.5	−9.9	−29.8	−63.1	−56.9	−41.8
southern and central N Sea	London	−29.4	−10.1	0	−1.7	−59.3	−106.7	−101.9	−72.3
	York	−28.9	−11.5	0	−15.8	−111.5	−144.5	−125.4	−71.8
Celtic Sea	Bristol	−41.8	−41.2	−8.9	0	−30.4	−217.3	−224.9	−116.3
	Cork	−38.7	−35.9	−16.3	0	−24.9	−212.4	−215.9	−120.8
western UK and Ireland	Launceston, Cornwall	−53.5	−77.0	−48.4	−16.5	0	−221.9	−206.2	−124.9
	Galway	−80.7	−99.5	−86.2	−21.1	0	−320.8	−346.1	−198.0
	Bornais, Outer Hebrides	−82.0	−105.8	−93.5	−38.1	0	−308.8	−313.2	−200.2
	Robert's Haven, Caithness	−53.9	−83.0	−69.0	−27.9	0	−245.7	−237.3	−150.4
Newfoundland	Dos de Cheval, Newfoundland	−171.5	−88.7	−172.7	−281.0	−498.4	0	−28.9	−74.6
Barents Sea and Iceland	Skonsvika, Finnmark	−82.4	−56.2	−104.7	−163.9	−270.5	−7.3	0	−32.7
	Kongshavn, Finnmark	−48.1	−52.1	−85.2	−139.7	−232.6	−39.7	0	−36.6
	Skriðuklaustur, Iceland	−121.8	−83.0	−151.2	−258.3	−457.5	−49.1	0	−56.7
eastern Baltic Sea	Gdańsk	−89.2	−57.3	−95.2	−136.3	−259.3	−57.0	−52.3	0
	Uppsala	−37.3	−40.4	−59.4	−91.7	−177.7	−61.7	−37.7	0

**Table 3.** AMOVA within and between the eight clusters of populations identified with BAPS.

source of variation	d.f.	sum of squares	variance components	percentage of variation
among clusters	7	376.17	1.26 (Va)	35.5 (*p*≤0.000001)
among populations within clusters	11	20.31	−0.03 (Vb)	−0.81 (*p*≤0.000001)
within populations	317	732.41	2.31 (Vc)	65.3 (*p*≤0.000001)
total	335	1128.89	3.54	

The samples generally group well spatially, yielding clusters or groups of clusters containing geographically close populations. The Caithness samples from northeast Scotland fall within the western UK/Irish cluster, differing significantly from the more proximate northern North Sea samples, indicating that there is a marked break in genetic population structure in northern Scotland which separates the western UK and Ireland from the North Sea. Interestingly, the Iceland samples fall within the same cluster as the Barents Sea samples using the selected SNPs, despite the relatively large geographical distance between the two. Furthermore, the Baltic samples appear to have the strongest genetic affiliation to Barents Sea/Iceland and Newfoundland, rather than to the North Sea group as might be expected from their geographical distribution and the genetic analysis of contemporary populations using purportedly neutral microsatellite genetic markers [43–45]. This indicates either a change in population structure over time or more likely the influence of selection on our set of SNP markers. The observed patterns of structuring reflect the levels of genetic drift and gene flow among populations, but also potentially divergent selection imposed by environmental gradients given that both neutral and selected SNP markers had been chosen for their power to discriminate between populations. Further analysis will be required to disentangle the fundamental evolutionary mechanisms that have created the observed pattern, but importantly these clusters are a robust framework for the purpose of assigning target samples to geographical populations.

### 3.3 Linear discriminant analysis of the isotopic baseline control data

As described above, the 33 location-based sets of isotopic control data were combined into five macro-groups that reflected historical cod fishing regions while maximizing successful reclassification. Evaluation of the final model using leave-one-out cross-validation gave reclassification success rates varying from 58.1 to 93.3%. The success rates for the Irish and southern North Sea (including the eastern English Channel) (75.5%), the eastern Baltic Sea (93.3%) and the very general region we define as Atlantic Europe (82.6%) are all high, whereas the discrimination of Newfoundland (60.0%) and Arctic Norway (58.1%) is less secure.

### 3.4 Genetic assignment of Mary Rose target samples to control populations

Of the 11 target samples from the Mary Rose ship, three were assigned to the northern North Sea, seven to the Barents Sea/Icelandic cluster and one to Newfoundland, indicating that all were fished in distant waters rather than being caught locally (figure 1*c* and table 4).

**Table 4.** Assignment of the Mary Rose target bones to the eight clusters using Trained Clustering in BAPS. Values are the change in log (marginal likelihood) if a sample is moved from its most likely cluster (logML=0) to a different cluster.

specimen	Celtic Sea	southern and central North Sea	western UK and Ireland	northern North Sea	eastern Baltic Sea	Lofoten and Bergen	Newfoundland	Barents Sea and Iceland
1	−30.2	−15.1	−43.9	−2.7	−10.5	−9.1	−19.8	0
2	−18.7	−11.6	−13.0	0	−32.0	−11.5	−48.4	−22.6
3	−51.9	−36.9	−78.7	−25.5	−14.8	−23.2	0	−5.5
4	−30.5	−23.8	−55.2	−11.1	−3.8	−14.7	−5.4	0
5	−43.2	−31.1	−70.1	−19.5	−0.3	−20.5	−9.3	0
6	−22.4	−11.5	−29.1	0	−12.3	−10.6	−21.5	−3.6
7	−52.2	−31.7	−84.6	−26.3	−5.1	−25.1	−4.7	0
8	−33.9	−23.7	−60.3	−16.5	−2.0	−12.0	−10.0	0
9	−63.9	−43.5	−92.6	−27.9	−11.0	−25.2	−6.8	0
10	−20.1	−16.3	−30.6	0	−12.9	−8.4	−22.3	−5.2
11	−28.5	−16.8	−40.5	−3.0	−10.2	−10.9	−10.4	0

### 3.5 Isotopic assignment of Mary Rose target samples to control populations

The *δ*^13^C and *δ*^15^N data for each specimen are presented in table 5, along with the probabilities of assignment to each region by LDA. The control and target stable isotope data are also plotted in figure 2*b*, allowing for visual comparison. Although outside the size range of the control data, the specimens from cod of more than 1000 mm TL do not plot separately from the other samples. On present evidence, the majority of the specimens are most consistent with origins in Atlantic Europe (a category that includes Iceland, northern Scotland and the Atlantic coasts of Ireland and southwest England) or Arctic Norway, with a single specimen (no. 3) more likely to be from Arctic Norway or Newfoundland. Regardless of their specific origin, none of the specimens have stable isotope values consistent with control data from the central and southern North Sea, the Irish Sea or the (eastern) English Channel. Moreover, previously published sulfur isotope data regarding the same Mary Rose samples are consistent with an offshore rather than inshore source [46]. All are likely to have derived from long-range trade or distant water fishing, though fisheries in the Celtic Sea or the western entrance to the English Channel remain a possibility based on the isotopic results alone.

**Table 5.** Stable isotope data, estimated total fish length (TL) and probability (*p*) of assignment to source for each of the Mary Rose target bones, using LDA (*p*-values of more than 0.25 are in italic). The abbreviations stand for: Atlantic Europe (AE, including Iceland, northern Scotland, western and southern Ireland and southwest Britain); Arctic Norway (AN, including the Barents Sea and the Norwegian coast as far south as the Lofoten archipelago); Newfoundland (NFLD); the Irish and southern North Seas (ISNS, including the eastern English Channel); and the eastern Baltic Sea (EB). The genetic assignments from table 4 are included for comparison.

specimen	aDNA assignment	estimated TL (mm)	*δ*^13^C	*δ*^15^N	C:N (molar)	AE (*p*)	AN (*p*)	NFLD (*p*)	ISNS (*p*)	EB (*p*)
1	Barents Sea and Iceland	876	−11.16	13.52	3.1	*0*.*9898*	0.0002	<0.0001	0.0100	<0.0001
2	northern North Sea	895	−11.02	13.82	3.1	*0*.*9789*	0.0001	<0.0001	0.0210	<0.0001
3	Newfoundland	851	−13.91	14.48	3.1	0.2180	*0*.*3971*	*0*.*2523*	0.1326	<0.0001
4	Barents Sea and Iceland	800–1000	−12.07	14.14	3.1	*0*.*9140*	0.0040	0.0012	0.0807	<0.0001
5	Barents Sea and Iceland	718	−10.59	13.91	3.1	*0*.*9791*	<0.0001	<0.0001	0.0208	<0.0001
6	northern North Sea	992	−11.44	14.21	3.1	*0*.*9269*	0.0006	0.0002	0.0723	<0.0001
7	Barents Sea and Iceland	800–1000	−13.30	14.00	3.1	*0*.*7389*	0.1372	0.0362	0.0877	<0.0001
8	Barents Sea and Iceland	794	−13.78	13.76	3.1	*0*.*5325*	*0*.*3600*	0.0654	0.0420	0.0001
9	Barents Sea and Iceland	¿1000	−13.72	14.16	3.1	*0*.*4390*	*0*.*3394*	0.1217	0.0998	<0.0001
10	northern North Sea	871	−11.06	13.55	3.3	*0*.*9895*	0.0001	<0.0001	0.0104	<0.0001
11	Barents Sea and Iceland	1015	−11.77	13.70	3.3	*0*.*9765*	0.0012	0.0002	0.0222	<0.0001

## 4. Discussion

Both the aDNA and stable isotope results suggest that the Mary Rose samples are of non-local provenance. Moreover, because each method uses different geographical groupings the genetic and isotopic evidences are complementary. The aDNA results exclude an Irish or southwest English source, which remained a possibility based on isotopic attributions to Atlantic Europe. Conversely, the assignment of seven samples to Iceland or Arctic Norway by the genetic evidence can be tentatively refined to Iceland in at least four cases (nos. 1, 4, 5 and 11), based on attribution of these samples to Atlantic Europe by stable isotopes. In three cases (nos. 7, 8 and 9, which cluster in figure 2*b*) either source remains possible; in these instances, the LDA results are split between Atlantic Europe (most probable) and Arctic Norway (second most probable). In other instances, the methods are broadly in agreement, corroborating the results. The attribution of sample numbers 2, 6 and 10 to the northern North Sea by aDNA is consistent with their assignment to Atlantic Europe by stable isotopes. The genetic attribution of sample number 3 to Newfoundland is also broadly consistent with isotopic LDA probabilities that are split between Arctic Norway (40%), Newfoundland (25%) and Atlantic Europe (22%).

Setting these results in historical context, both Iceland and the waters of northern Scotland were known sources of dried cod (with and without salting) in the sixteenth century [20,22]. Arctic Norway had ceased to be a major supplier of the English market by this date, but remained an important source on the Continent, opening the possibility of supply by middlemen, and occasionally English fishermen themselves may have worked in northern Norwegian waters [47]. Our genetic data further indicate that one sample was probably sourced from even more distant fishing grounds off the North American coast. Unfortunately, the isotope data could not provide the resolution to confirm or refute this hypothesis. Nevertheless, the English Newfoundland fishery had begun in 1502, in the wake of John Cabot's exploratory voyage of 1497 [21], making this entirely plausible.

Regardless of the origin of this last intriguing specimen, all of the aDNA and stable isotope evidence is consistent with the interpretation that the preserved cod used to provision the Mary Rose were ultimately sourced in the north or from across the North Atlantic, far from Portsmouth from which she sailed, and that the fish were drawn from diverse sources. Given that dried fish were served as the main naval ration for three of every seven days at this time [48], it is reasonable to infer that military needs contributed to the demand for cod from distant waters. In comparative context, the requirements of warfare influenced the growth of domestic fisheries in sixteenth century Scandinavia [49]. In the reign of Henry VIII (AD 1509–1547), English demand may still have been quantitatively modest; the Anthony Roll records that the royal fleet totalled only *ca* 58 vessels in the 1540s [50]. Nevertheless, a navy had become a royal priority, and during the reign of Elizabeth I (AD 1558–1603) it proved essential in competition with increasingly global powers such as Spain [10]. A link between the English fishery and navy was made explicit during Elizabeth's reign by the introduction of weekly ‘fish days’ to encourage domestic consumption and thus a commercial fleet [51]. This legislation aimed to ensure a supply of trained mariners, but must concurrently have promoted a secure source of provisions. The importance of victualling the navy continued to grow in the seventeenth century, most famously during the Restoration when its administration was systematized under Samuel Pepys [52]. Military sea power was a prerequisite for the concurrent (and subsequent) development of England's sea-borne colonialism, with its well-known consequences for terrestrial ecosystems globally [4]. Yet by sourcing the cod bones from the Mary Rose, we see that the navy itself was first sustained, in part, by fishermen working distant northern and potentially transatlantic waters. Thus, the commercial exploitation of aquatic ecosystems and the growth of sea power were mutually reinforcing aspects of globalization in Renaissance Europe.

## Supplementary Material

Figure_S1

## Supplementary Material

Figure_S2

## Supplementary Material

Table_S1

## Supplementary Material

 Table_S2

## Supplementary Material

Table_S3

## Supplementary Material

Table_S4

## Data accessibility

The supplementary material includes: table S1 Control samples of cod bone used in the study and stable isotope data; table S2 Kruskall–Wallis test results for temporal differences in *δ*^13^C and *δ*^15^N values within the archaeological reference dataset, by geographical macro-group and two-century block; table S3 PCR primers used in the four multiplex reactions; table S4 SNP genotypes for the control and target samples.
