# Supplementary figures and images for: The globalization of naval provisioning: ancient DNA and stable isotope analyses of stored cod from the wreck of the Mary Rose, AD 1545
Source: R Soc Open Sci. 2015 Sep 9;2(9):150199. doi: 10.1098/rsos.150199 (PMC4593681; doi:10.1098/rsos.150199)

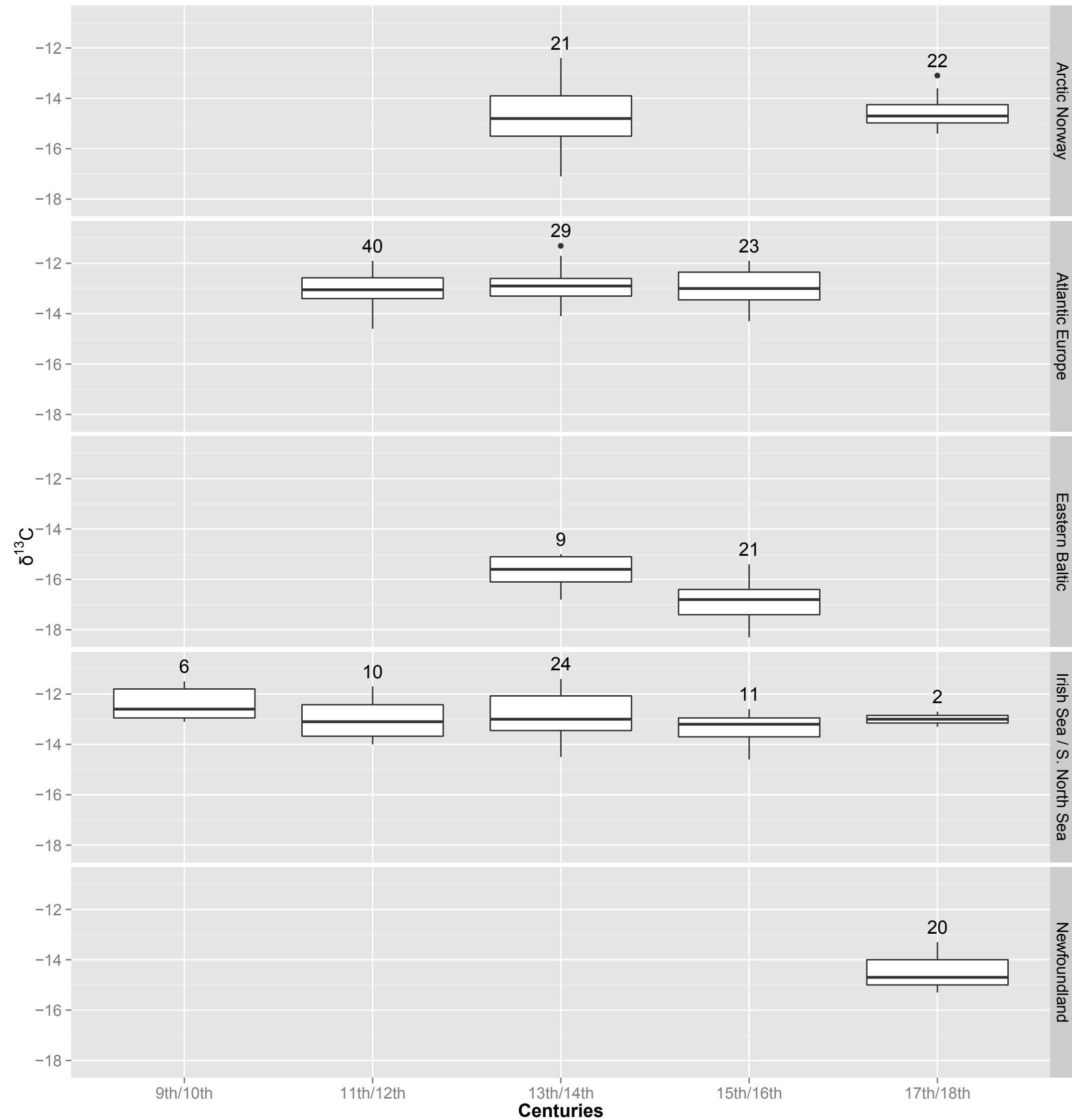

Supplement: Figure_S1 [file rsos150199supp1.pdf]

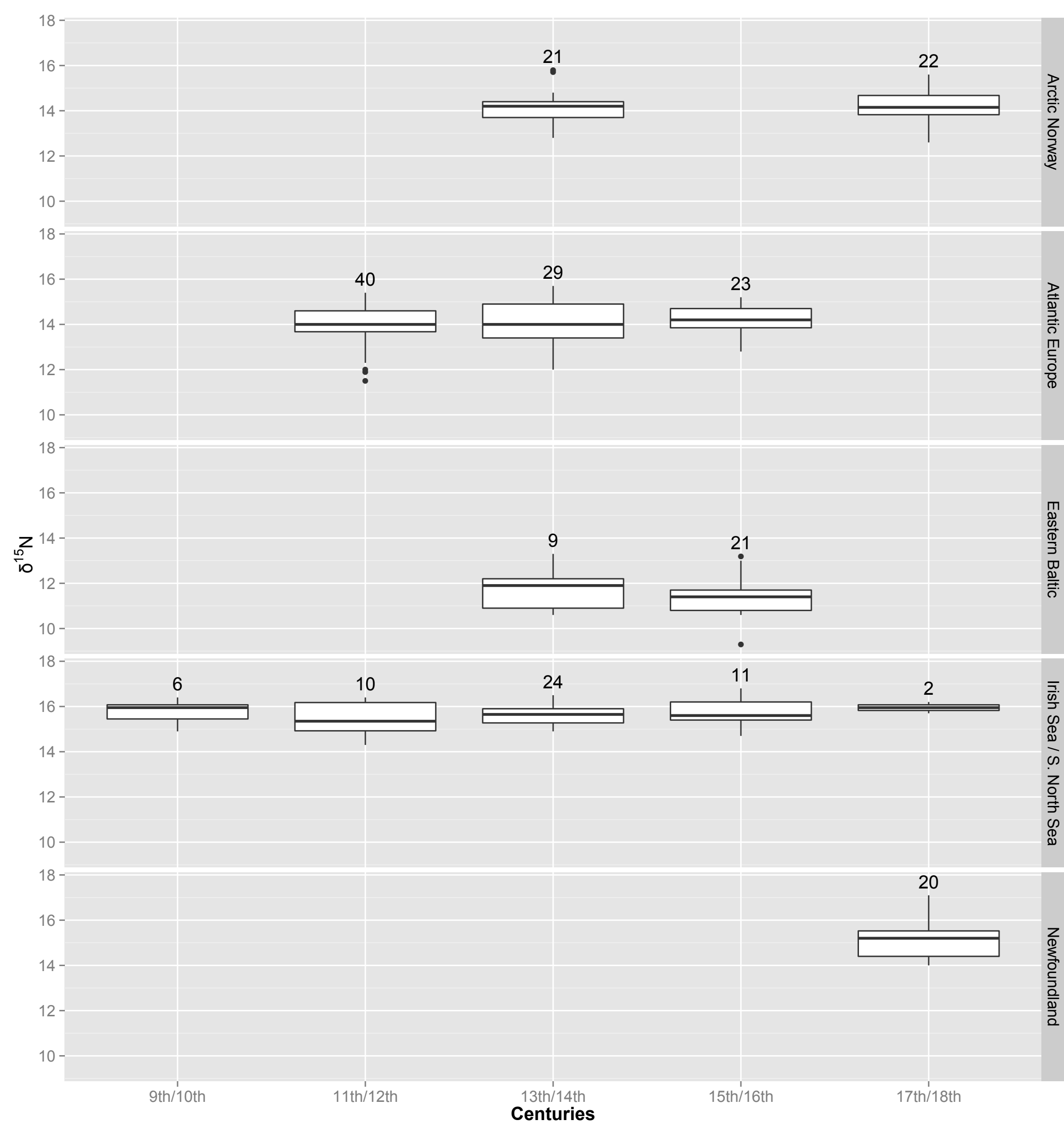

Supplement: Figure_S2 [file rsos150199supp2.pdf]
